# Development of prehospital emergency care in Singapore

**DOI:** 10.1186/s12245-023-00582-1

**Published:** 2024-01-22

**Authors:** Gayathri Devi Nadarajan, Nurul Asyikin Binte Mohamed Jalil, Alexander Elgin White, Marcus Ong Eng Hock, Anantharaman Venkataraman

**Affiliations:** 1 Department of Emergency Medicine, Singapore General Hospital, Singapore, Singapore; 2 Unit for Pre-Hospital Emergency Care, Ministry of Health (UPEC), Singapore, Singapore; 3 Duke-NUS Academic Medical Centre, Emergency Medicine Academic Clinical Program, Singapore Health Services, Singapore, Singapore

**Keywords:** Emergency medical services, Out-of-hospital cardiac arrest, Pre-hospital emergency care, History

## Abstract

This review paper describes the development of the pre-hospital system in Singapore from the pre-war days. Every country’s prehospital community needs a deep understanding of how they developed over the years, factors that played a part, and the aspirations their community and government have set for this. This can guide future evolution of the services to ensure that care provided is relevant, applicable and in keeping with the community’s needs. Countries with similar contextual circumstances, but at a different stage of development of their PECs, may learn from these.

## Introduction

Development of pre-hospital emergency care (PEC) in a country is often dependent on individuals who captained such systems. Some learn systems prevalent in mature economies and use these as a basis for setting up their own. Most use variations of either the Anglo-American or the Franco-German model of prehospital care. Increasingly, with global health, we have seen teams from resource-rich countries preach the virtues of their systems to less endowed communities. The eventual organization, structure, and methods of operation of prehospital systems in the newer economies have resulted from the combination of these influences, combined with the host community’s resources and organizational relationships within that country.

Every country’s prehospital community needs a deep understanding of how they developed over the years, factors that played a part, and the aspirations their community and government have set for this. This can guide future evolution of the services to ensure that care provided is relevant, applicable, and in keeping with the community’s needs. Countries with similar contextual circumstances, but at a different stage of development of their PECs, may learn from these.

Singapore, a cosmopolitan city-state located in South-East Asia just off the southern-most tip of the Asian continental land mass and just 725 km^2^ land area is one of the smallest countries in the world. Its population of 5.7 million has a multi-ethnic mix of Chinese, Malay, Indian, Europeans, Eurasians, and others [1]. The health services, including PECs, recognize these variations in trying to meet the challenges of providing emergency care to this heterogenous population. The bulk of prehospital emergency medical care in Singapore comes under the umbrella of a public organization, the Singapore Civil Defence Force (SCDF) that is managed, not by the Ministry of Health (MOH), but by the Ministry of Home Affairs (MHA). In 2019, the SCDF’s Emergency Ambulance Service (EAS) operated a fleet of 80 ambulances and responded to 191,468 emergency ambulance calls.

This report traces the evolution of the services from its beginnings and documents some of the challenges along the way, especially in three areas, viz. organizational system, ambulance care practices, and non-ambulance-based areas such as disaster management and emergency life support. Since these evolved spontaneously, without a pre-determined long-term developmental direction, they will be described chronologically, rather than based on the individual system so as to better understand how each has kept pace with the others. It incorporates initiatives implemented to provide the best level of care that members of the community deserve. Though regarded as first-world now, these lessons may be useful for countries attempting to evolve their own systems.

### The report

Information for this report was gathered from various sources. The Singapore National Archives was contacted for information pertaining to pictures of old casualty transportation vehicles. PubMed and Google Scholar were sourced to obtain copies of publications that touched on the local pre-hospital emergency services and related areas. Interviews with current and previous leaders of the EAS, disaster medicine thought leaders and life-support pioneer leaders in Singapore helped address areas for which publications were not available. Historical annual reports and publications of local healthcare agencies were also reviewed for additional relevant information.

### 1917: the beginnings

**Fig. 1:**
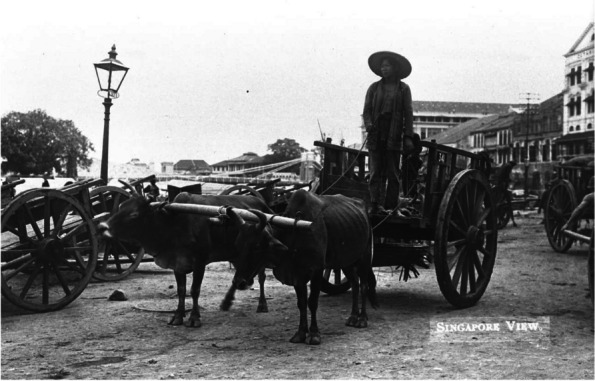
Bullock cart (Singapore, 1910) © National Archives Singapore

**Fig. 2:**
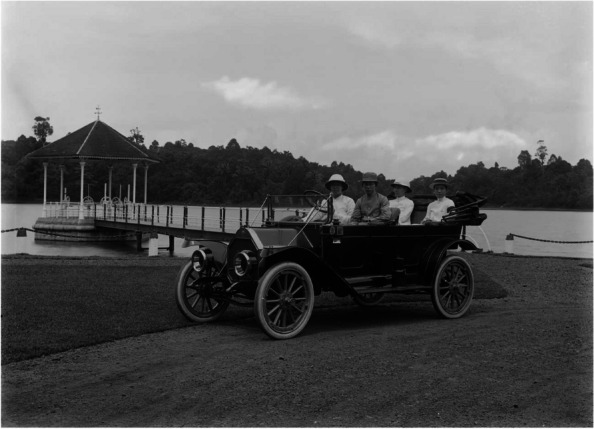
A motorcar used in Singapore in the early twentieth century ©National Archives Singapore

**Fig. 3:**
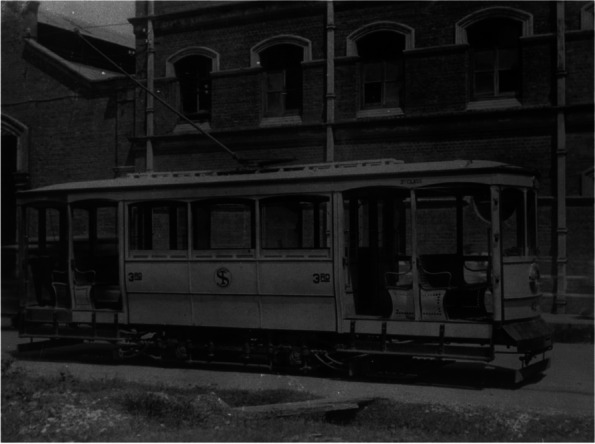
Tram car (Singapore, 1900s) ©National Archives Singapore

Very little is known about the state of ambulance services prior to 1917. In 1886, the St John Ambulance Association provided first-aid training to the Police [2]. In 1890, a police ambulance provided first aid and evacuated injured jockeys to the Singapore General Hospital [3]. Prior to 1917, patients were brought to hospitals by any available transport such as bullock carts, two-wheeled horse-drawn carriage or four-wheeled horse-drawn carriages (gharries) which brought an injured ship officer from the harbor to the General Hospital in 1901 [4], runner-pulled jinrickshaws, motorcars, and even trams (Figs. 1, 2 and 3). The first record of an accident involving an ambulance was in 1906 when a horse-drawn ambulance collided with a car resulting in injuries to the ambulance occupant [5]. In April 1913, a horse drawn carriage was used as an ambulance by the local municipal council. The government of the day had then raised the matter of acquiring a motor ambulance [6].

On 10 September 1914, the Straits Times reported that a drowned victim was resuscitated by a St John trained first-aider after artificial respiration for about 20 min [7]—the first documented instance of a patient with out-of-hospital cardiac arrest in Singapore being saved by a trained member of the public with artificial respiration.

The roots of an organized motorized ambulance service can be traced back to 1917 when a motor vehicle was presented to the Singapore General Hospital (SGH) Board but placed under the care of the Singapore Fire Brigade (SFB) at the Central Fire Station (CFS) because the hospital was undergoing major renovation. This ambulance was staffed by workers from the SGH and transported mainly trauma patients to the hospital. From 1917 to 1927, the number of calls to the trauma ambulance service increased from 57 to 1358 per annum.

In 1927, a member of the then Singapore Legislative Council, Mr. Song Ong Siang, proposed that the government have one ambulance garaged at the SGH and one at the Tan Tock Seng Hospital (TTSH) [8]. Eventually one was located at the CFS and the other at the Central Police Station. In 1928, two other trauma ambulances were added to the SFB and located at the CFS which was in the city center. Retired firemen manned these ambulances. No formal treatment was provided to the casualties. The ambulances were used mainly as transportation vehicles.

### The war years: 1942–1945

Just prior to the aerial bombing of Singapore by the Japanese in late 1941 and early 1942, a Medical Auxiliary Service (MAS) was set up by the government with first-aid posts in various buildings around the island manned by first-aiders from the St John’s Association and Brigade and reinforced by volunteers assisted by over 200 students from the King Edward VII College of Medicine. Many worked long hours during the height of the attacks. During the aerial bombing by the Japanese and up to the surrender by British Forces in February 1942, the dead and injured were transported by any available vehicle, to the SGH and the TTSH. No information is available on what happened to the ambulances during the Japanese occupation. The recovery period following the Japanese surrender in August 1945 saw the ambulances returning to their pre-war role, viz. transporting trauma patients. The MAS, however, was not resuscitated.

### The immediate post-war years: 1946–1964

The return of the British after the Japanese surrender was a tumultuous period for Singapore. The SFB’s Ambulance fleet was enhanced to 11 vehicles.

In terms of training, the Singapore Red Cross Society (SRCS), which was set up in 1949, provided assistance to the SGH ambulance crew in its early years. First-aid instructors from the St John Ambulance trained ambulance crew of the Police and SFB ambulances.

With increasing demand for non-trauma medical evacuations and insufficient ambulances, the SRCS offered help by ferrying patients to the hospital in their own ambulance. In 1968, with the expansion of SRCS to incorporate nursing auxiliary services in hospitals, a Night Ambulance Service (NAS) operational from 7 pm to 7am daily was begun. This service was provided by the Voluntary Aid Detachments (VAD) of SRCS and attracted many volunteers. By the end of 1968, there were 1127 volunteers registered with the VAD across 14 detachments. In 1969, the SRCS Ambulance Service became a supplementary service to the official ambulance service provided by the SFB. In the 1970s, the NAS was discontinued as the SFB expanded its ambulance unit. The SRCS and St John Ambulance Services became private ambulance services and were the first of many more private agencies that set up non-emergency ambulance services which now total more than 10.

Institution of emergency rule by the British from the late 1940s until the late 1950s saw the occurrence of terrorism-type incidents in the region beginning with the Malayan Emergency in 1948. It was during this very tumultuous period that the first Emergency Department (ED) was started in Singapore at the SGH in 1948 [9]. In 1950, mass racial riots occurred with casualties who required evacuation. There was no concept of disaster-site medical management then. Casualties were either loaded onto any available vehicle or into SFB ambulance vehicles and transported to the SGH. It was a simple scoop-and-run system. The ambulances were ill-equipped to provide any major stabilization of casualties. Similar situations were encountered during political riots that occurred in the mid-1950s.

In 1954, a British Airways flight crashed at the Singapore Airport resulting in many casualties. An ambulance rushing some survivors to the SGH was involved in a collision with a lorry, and the patients were transferred by police radio cars to the hospital [10]. The incident was a wake-up call not only on the need to plan for ambulance response in disasters, but also in the areas of ambulance and crew safety.

### 1964–1977: merging of central and trauma ambulance services into emergency ambulance services

A non-trauma central ambulance service coordinated by SGH was set up in 1964. The trauma ambulance service stayed with the SFB. There was still no single ambulance call number for members of the public in the event of an emergency. The average waiting time for a central ambulance which was staffed by nurses was about 25 min [11]. The SFB trauma ambulances had an average waiting time of about 15 min. This period was typified by lack of dedicated or trained ambulance crew and inadequate ambulance equipment. The fire brigade did not have medically trained staff. There was also a separate maternity ambulance service operating from the Kandang Kerbau Hospital, which was then one of the largest and busiest maternity hospitals in the world [12].

To address the wide variations in the quality of emergency ambulance services being provided, in 1977, these three services were brought together under the SFB to form a single public Emergency Ambulance Service (EAS). The MOH provided 84 nurses with midwifery training. Additional ambulance crew from the SFB were also added to the EAS. The nurses underwent a 2-week orientation at the SGH ED before being posted to the EAS. The EAS had 18 ambulances, each with one trained ambulance officer (nurse), one young 18-year-old performing mandatory national military service, and one ambulance driver, all of whom were given first-aid training. Splints, bandages, dressings, and maternal delivery sets were also provided in the ambulances. The only medications available were Entonox, morphine (for which the nurses required the permission of an ED doctor to use in every instance), and oxytocin for delivery cases. There was no formal system of medical oversight for these ambulances. By 1977, there were 35,819 patients using the EAS service and this increased to 64,312 in 1979.

### 1978 to 1988: disasters, emergency medicine and a single call number

In 1978, the EAS was called upon to mobilize almost all its ambulances to support a major fire disaster, at a major shipyard in the country. This was the first major test of rapid mobilization and disaster support for the EAS. The St John Ambulance and the SRCS also supported the casualty evacuation efforts.

In the 1980s, four major events helped shape the future development of emergency care and the prehospital service. Firstly, cardio-pulmonary-resuscitation (CPR) training was introduced into the EAS in 1983. Then, in 1984, the MOH gave official recognition to emergency medicine as a medical specialty. This led to the development of structured post-graduate training programs and regular formal teaching in emergency medicine. These trained emergency physicians would subsequently help to take prehospital care in the country to greater heights.

In the same year, a universal access number was given to the EAS (995) to replace the older numbers such as 5555, 328,111, and 3,378,111. The availability of a three-digit number for ambulance access led to increasing number of ambulance calls. Non-emergency ambulance requests were co-ordinated by a four-digit number, 1777.

The collapse of a building, the “Hotel New World”, in 1986 resulted in more than 30 fatalities and demonstrated the poor state of preparedness of public hospitals in Singapore to provide on-site disaster medical care. This resulted in a review of disaster response procedures and in a new civil disaster medical response model consisting of a quick-response disaster site medical command consisting of medical teams from all public hospitals with standardised equipment and reinforced, if needed, by similar teams from the public primary healthcare services and the armed forces. The revised health services disaster response system has been used in Singapore since then to good effect for a number of local incidents occurring in our shipyards, airports, ferry terminals, and other locations.

### 1989 to 1995: a new parent and speeding up ambulance response

In 1989, the SCDF took over command and control of the SFB, including the EAS. The motto, “A Nation of Life Savers” was adopted by the service to emphasize that their work was not just about fire-fighting and rescue but also pre-hospital medical care.

In that same year, automated external defibrillators (AEDs) were introduced to two emergency ambulances of the SCDF and saw their first save within two weeks of the launch of the scheme. In 1995, AEDs were emplaced on all SCDF ambulances and the Heart Save project to improve the ambulance service’s management of out-of-hospital cardiac arrest was born.

In 1990, training of all nurses in the EAS in the skills of Basic Trauma Life Support (BTLS) was started. To respond faster to trauma emergencies, especially during difficult traffic conditions, a motorcycle-based fast response medic (FRM) scheme was introduced in 1992. With the focus on immediate trauma care, training in BTLS was provided to the FRMs. Three years later, AEDs were added to the motorcycles to enhance the response to cardiac arrest patients. This was a first for Asia. Patients attended to by the FRMs were subsequently transported to the hospital by ambulances. This scheme reduced the response time from call to arrival by the patient’s side from a mean of 15 to about 8 min [13]. Together with other measures that have been implemented concurrently such as public CPR + AED training and telephone CPR, these have resulted in significant improvements in survival rates for out-of-hospital cardiac arrests.

### 1995–2010: paramedics and medical oversight

In 1995, a joint review of the ambulance services by the MOH and MHA was conducted as a result of realization that the amount of care being provided by the ambulances was limited to mainly basic first aid and some CPR and use of AEDs and concern that the national nursing shortage was impacting the provision of trained staff in the emergency ambulances. This resulted in a move towards a locally relevant and treatment-orientated paramedic-based system. Two committees were set up—the Medical Advisory Committee (MAC) and the Paramedic Training Committee (PTC) [11]. A medical department headed by a chief medical officer was established within the SCDF to oversee the operations of the EAS. The Armed Forces Military Medical Institute was tasked to house and oversee conduct of the training of paramedics in the country.

The MAC was headed by an emergency physician and consisted of two other emergency physicians, one representative each from the General Surgery, Cardiology, Orthopaedic Surgery, Anaesthesia and Paediatrics, the Chief Army Medical Officer, and the Chief Medical Officer of SCDF. The MAC adopted the Anglo-American model and created standardized and Singapore-specific emergency ambulance care protocols, emergency drug monographs, and formal clinical procedures for the EAS. Additional drugs for use within the EAS included aspirin, adrenaline, nitroglycerin, hypertonic dextrose, and salbutamol. Additional procedures included use of laryngeal mask airways, intravenous cannulation, acquisition, and transmission of 12-lead electrocardiograms. The introduction of structured emergency ambulance treatment protocols (34 in number) and a clinical quality management system with retrospective audit resulted in the start of indirect medical oversight. The MAC also introduced training of medical dispatchers in the SCDF to not only fine-tune the dispatching of available emergency ambulances, but also provide treatment advice, such as telephone CPR and advice on care of wounds, to those calling for emergency ambulance assistance. During this 15-year period, the ambulance fleet increased in size to 30 ambulances and reinforced by 10 ambulances from the private sector working under the operational control of the EAS. To address the need for more ambulance crew, the MAC accepted proposals from other tertiary institutions in the country that had interest in the training of paramedics, such as the Institute of Technical Education and one of our polytechnics, to train more ambulance crew and increase the complexity of training programs to enhance knowledge and skills of the crew. The nurses seconded from the MOH were gradually replaced by trained paramedics. The ECG transmission system introduced helped decrease door-to balloon times for myocardial infarction patients in all public hospitals from 75 to 51 min [14].

The PTC was headed by the Chief of the Medical Corps of the Singapore Armed Forces (SAF) with MAC Chairman sitting in as a member. The training needs were usually identified by the MAC. These needs would be sent to the PTC for implementation. Trainers came from the armed forces and EDs of public hospitals. This arrangement ensured a close collaboration between SCDF, public Eds, and the SAF that provided the training facilities for the paramedics. With the need to address concern that the quality of ambulance crew training was on par with those in first-world countries, accreditation of the training conducted by the military medical institution was sought and obtained from a well-established training provider chosen because of similarities in ambulance-based clinical care, viz. Canada’s Justice Institute of British Columbia, after a rigorous accreditation exercise. Later programs developed by the SAF Military Medicine Institute included the Diploma in Health Services (Emergency Medical Services) and Primary Care Paramedic Programs.

Communication with hospitals is necessary for ambulance operations. Conventional methods of verbal communication, including high frequency radio and cellular telephones were continued. Verbal communications had their limitations such as variable voice quality, the need for written records and the technical difficulty of capturing and transmitting a range of clinical information. To improve data collection and communications, the MOH, National Computer Board, SCDF, and the SGH together developed a wireless information technology system to supplement existing voice links between the ambulance crew and the EDs in 1998. The result was the Hospital and Emergency Ambulance Link (HEAL), which was piloted on three ambulances [15]. HEAL had a user-friendly client server application with features such as touch screen and “canned” text to facilitate data entry. Mobile computers in the ambulances automatically captured vital signs and forwarded these to the receiving hospital via a wireless communications network. This information together with biodata, clinical and patient management information, created a complete electronic prehospital record.

HEAL’s main objective was to facilitate early patient care in EDs. It also enhanced documentation. A 3-month pilot run of HEAL revealed the possibility to capture complete ambulance case record electronically within 2 min versus 7 min for the traditionally written record. In addition, much more data was transmissible to the receiving ED compared to non-HEAL ambulances. Time spent by paramedics in the ED after handing over the patient to ED staff and ED waiting times for critical care patients was reduced by half. HEAL was also able to prompt paramedics to carry out critical aspects of treatment in all instances and demonstrated the feasibility of electronic data collection in the prehospital environment. However, at that time, it could not be deployed nation-wide owing to prohibitive costs.

In 1998, MOH launched the National Resuscitation Council (NRC) to support the teaching and practice of resuscitation life support programs, act as a coordinating body representing major groups involved in the teaching and practice of resuscitation, promote uniformity and standardization in resuscitation practices, and provide direction, national policies and guidelines for the teaching and practice of resuscitation. The NRC encouraged the setting up of life support training centres in the country. One of the largest of these was at the SCDF Training Academy. The EAS paramedics were some of the most active instructors in CPR and defibrillation and trained thousands in these vital life-saving skills. The community programs pushed by the NRC, introduction of annual National Life Saving Days, promotion of CPR + AED training programs for the public since 2005, and the piloting of AEDs in public housing estates with training in these skills provided near their places of residence in 2008 all helped to push the numbers of Singapore residents trained annually in these skills to about 180,000 persons per annum in 2017. These also resulted in an increase in the bystander CPR rate from 21% in 2002 to more than 60.9% in 2020^/^ [16] and contributed to an improvement in the rate of survival of out-of-hospital cardiac arrest patient at hospital discharge from 2.0% in 2002 [17] to 25.9% in 2018 [18].

In early 2009, SCDF together with the MAC introduced a Medical Priority System for ambulance dispatch to better prioritise resources used for different types and levels of cases that call the SCDF. The system included provision of pre-arrival instructions to the callers. Hands-only CPR instructions for out-of-hospital cardiac arrest was introduced into the SCDF Dispatch protocols later in the year. This was one of the factors that contributed to the increasing bystander CPR rates.

### 2010–2020: creating the future pre-hospital emergency care system

In 2010, the SCDF EAS introduced the Smart Pen, after having piloted it a year earlier. This has allowed paramedics to digitally capture patient data as they are being written into the ambulance case record forms and has simplified the process of conducting audit of ambulance runs.

In 2011, mechanical CPR was piloted in a limited number of SCDF ambulances. The pilot demonstrated doubling of rates of survival to hospital discharge for out-of-hospital cardiac arrest if the device was started at site [18]. Its use has since been extended to all emergency ambulances.

In 2011, a Unit for Prehospital Emergency Care (UPEC) was established at the SGH by both MOH and MHA to assist in provision of medical oversight and systems decision support for enhanced prehospital emergency care. UPEC coordinates, monitors, and implements various PEC plans and works closely with MOH, SCDF, and other stakeholders involved in the training of PEC professionals, private ambulance operators (PAO), hospitals, and community partners. UPEC focuses on 6 domains, viz. medical operations and oversight, information technology, prehospital emergency care research and policy, professional training and education development, standards and licensing, and community responsiveness. UPEC has paved the way for collaboration with neighbouring countries through its pre-hospital overseas fellowship programs, medical director’s workshops and participation in international organizations. UPCE has rolled out a number of programmes to improve quality of prehospital emergency care [19]. One was in 2013 when the Dispatcher-Assisted first REsponder (DARE) program which teaches participants how to recognize cardiac arrest and call for an ambulance was introduced. In 2015, UPEC with SCDF developed a National Paramedic Training and Education Roadmap to enable paramedics to advance in their careers further by completing an advanced diploma or bachelor’s degree in paramedicine. This was implemented in 2021. UPEC also established the Medical Director’s workshop for both EAS and other private medical transport service providers, guidelines for oversight, standards of professional competencies of various levels of prehospital emergency personnel, and accreditation of foreign-trained paramedics for employment in Singapore.

**Fig. 4:**
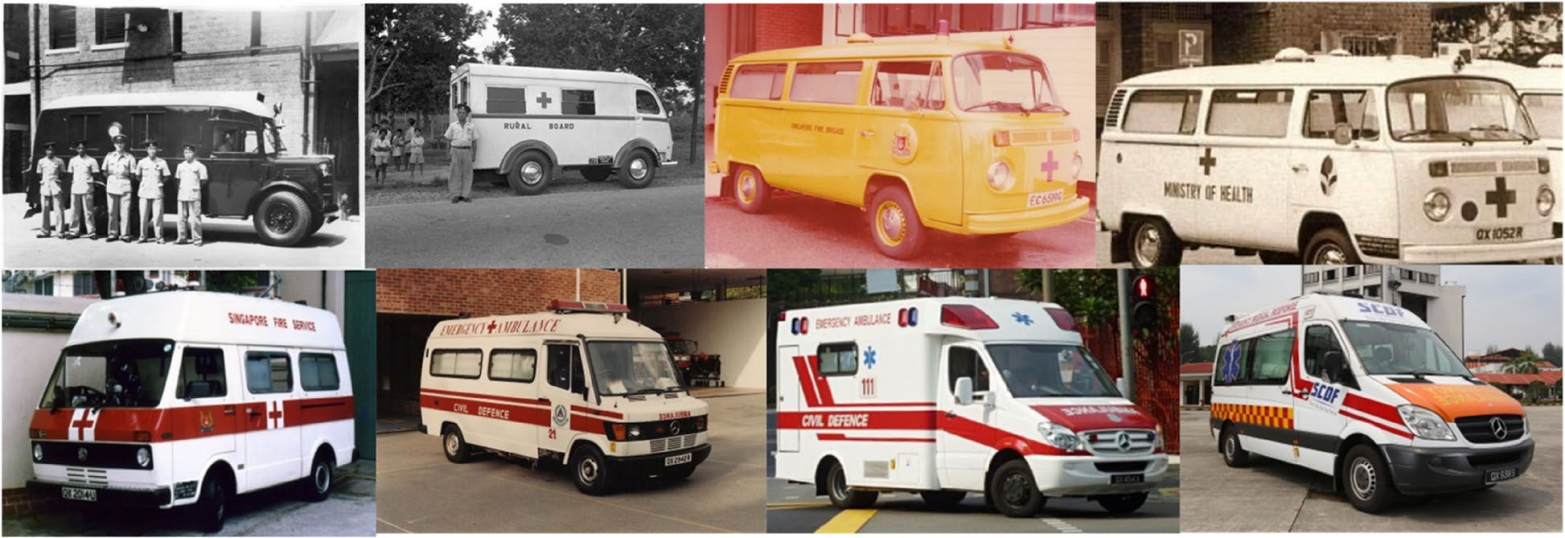
Evolution of our ambulances (from top left, clockwise; Austin Sheerline (1935), Austin K9 Welfarer (1948), Volswagen Transporter (1959), Volswagen Transporter (1960s–1970s), Volswagen LT31 (early 1990s), Mercedes Benz (1993), Mercedes Benz Sprinter (2009), and Mercedes Benz Sprinter (2015)

The SCDF Medical Department has been significantly reorganized since 2012 with the doubling of the EAS fleet from 40 to 80 ambulances by 2019, a shift from American style box-car ambulances (Fig. 4) to European style van chassis ambulances for increased safety, creation of a dual fleet ambulance strategy (to provide a spare vehicle for every ambulance on the road and to provide surge capacity). Ambulance drivers were upgraded to EMTs, and firefighters were cross-trained and cross-deployed as firefighter-EMTs on fire bikes initially and progressively to other fire appliances. The SCDF Medical Department added three sections into its organization, viz. data analytics, quality audit, and medical projects. An administrative director was appointed. Since 2014, nurses have been seconded from hospitals to the SCDF operations centre to assist dispatchers during a cardiac arrest call [20].

A handphone application called “myResponder” was also created by the SCDF to notify Community First Responders of potential cardiac arrest cases within 400 m and show the location of AEDs by linking to a Registry for AED Integration (R-AEDI) [21].

In 2015, collaboration was initiated between SCDF, UPEC, the Singapore Heart Foundation, and the Peoples’ Association (a community grassroots organization) to improve community first response to cardiac arrest with conduct of DARE programs in the community and future installation of AEDs in every alternate high-rise public apartment building (in which 85% of the population reside) island-wide.

In 2017, a Healthcare Services Act for EAS and other MTS providers was enacted to provide clear guidelines for more effective management of these private ambulance operators and new licensing requirements for ambulances.

Online medical direction was launched in 2018, where paramedics are able to get phone advice from Emergency Medicine Specialists from various public hospitals.

In 2019, SCDF began dispatching fire appliances together with ambulances for adult cardiac arrest cases to improve response times and for the conduct of high-performance CPR with the intent to improve survival. The impact of this intervention is currently being reviewed. The EAS has also rolled out “treat and discharge” protocols to guide paramedics on management of patients who would not require conveyance to hospital.

In 2020, the EAS began working with UPEC to develop an ambulance simulation and deployment optimization system to further reduce ambulance response times. The EAS also developed an electronic ambulance record system along the lines of the HEAL system. Called OMNII (Operational Medical Network Informatics Integrator), it was launched in late 2021 to help integrate information flow between the EAS and hospital EDs, thereby facilitating more seamless care. Paramedics will be able to view selected patient hospital records on a handheld tablet, pre-register the patient, and prepare the ED for the patient’s arrival.

For the near future, UPEC is looking into use of artificial intelligence to more rapidly triage emergencies, reduce time-to-dispatch, and ensure appropriate resource use to match patient severity. It is also looking at Alternative Care Service Pathways (ACSP) to right site patient care within the community instead of conveying them to the already overloaded hospitals. This is in line with the country’s growing problem of an ageing population needing more support in the community and having longer stays in the hospitals.

## Conclusion

Pre-hospital emergency medical care in Singapore has involved the development of organized ambulance services, first-aid and life-support training systems, and practice and healthcare support during disasters and mass casualty events. These have progressed remarkably and continue to evolve. This has been a result of collaboration with various governmental and community organizations. The development has taken time. Other countries can learn from our experience and journey. Many countries have different systems, demographics, and circumstances. Adaptability needs to be considered. Being a small country, it has been easier for Singapore to implement changes over relatively shorter time periods and study the effects of these.

## Data Availability

All relevant data are within the manuscript.
